# Address Delivered at the Opening of the New Clinical Lecture Room of the Philadelphia Hospital

**Published:** 1862-01

**Authors:** 


ADDRESS Delivered at the Opening of the New Clinical Lecture Room of the Philadelphia Hospital.—By Dr. J. L. LUDLOW, one of the Medical Board and Lecturer on Clinical Medicine. Philadelphia: 1861. 8 vo. pp. 26.

An attempt to give anything like a satisfactory "general account of the rise and progress of Clinical Instruction," within the limits of an introductory lecture, might be reasonably anticipated to end in disappointment; and we must confess to this feeling on laying down Dr. LUDLOW's *Address.* But surely, we had a right to look for something interesting and instructive where the theme itself was so suggestive and important; we might expect, at least, tolerable grammar and correct orthography from a lecturer in a Philadelphia school, —"Philadelphia," where "has been erected that medical reputation and renown, which has eclipsed all the other cities on our continent, and made Philadelphia, (which may she long continue to be,) the metropolis of medical science in the western hemisphere."*

Dr. LUDLOW has done himself injustice in allowing, what are evidently nothing but the rough notes of his address, to be handed to the printer, and thence set before the profession, without his revision, in even so vital a matter as the connection of the component clauses of a sentence, as in the following:—

"Notwithstanding the American Colonies boasted several

---

* Page 19, Dr. LUDLOW's *Address.* ("Which she were that good in her heart," as *Joe Gargery* says.)

medical characters of note, and as early as 1750, the body of a criminal, Harmanus Carroll, executed for murder, was dissected, and the blood vessels injected for the instruction of medical students, by Drs. John Bard and Peter Middleton of New York. (The *first recorded* dissection of the human body for medical purposes in this country.) And although in our own city lectures had been delivered, yet no systematic course of medical instruction was given in this country until 1765, when Drs. Shippen and Morgan, delivered the first *course* of lectures in this city."

Of course, Dr. L. would not have allowed this to stand uncorrected, had he seen the proof;—nor "educed" for *adduced* on the following page (17), ninth line from the bottom; nor "breech" for *breach*, page 24, second line; the substantive "wreaths" for the verb *wreathes*, same page, sixteenth line, and other similar errors, too numerous to be excusable as mere oversights—for very evidently, he did not see either them, or their context, after the manuscript had passed from his hands.

The following extract proves that Dr. Ludlow can write correctly, and that the *materiel* is not wanting for an interesting discourse on the subject he had chosen, in place of the illy-connected jumble of dry dates and odds-and-ends he has caused to be printed:

"And now, gentlemen, we have arrived at that epoch in the history of medicine in our country, when *the first Clinical lecture* was delivered in America. In the Pennsylvania Hospital, on the 3d of December, 1760, Dr. Thomas Bond, one of the physicians to that institution, gave the introductory Clinical discourse.

"I cannot forbear quoting from this lecture, if not for instruction, nevertheless from curiosity, that we at this late day may see how the father of clinical instruction in America appreciated the advantages which must necessarily arise from a proper clinical course.

"Speaking of Dr. Morgan, the Professor of Theory and Practice in the University, he remarks:—'The field this gentleman undertakes is very extensive, and has many difficulties, which may mislead the footsteps of an uncautioned traveller. Therefore, lectures in which the different parts of the theory and practice of physic are judiciously classed and systematically explained, will prevent many perplexities the student would otherwise be embarrassed with; will unfold the doors of knowledge, and will be of great use in directing and abridging his future studies. Yet there is *something further wanting:* he must *join examples with study*, before he can be sufficiently qualified to prescribe for the sick, for *language* and *books alone can never* give him adequate ideas of diseases, and the best method of treating them; for which *reasons Infirmaries are justly reputed the grand theatres of medical knowledge.* There the *Clinical Professor comes to the aid* of speculation, and demonstrates the *truth of theory by facts*, etc., etc.' Further on again he says: 'I am now to inform you, gentlemen, that the managers and physicians of the Pennsylvania Hospital, on seeing the great number of you attending the School of Physic in this city, are of opinion that this excellent institution affords a favorable opportunity of further improvement to you in the practical part of your profession; and being desirous it should answer all the good purposes intended by the generous contributors to it, have allotted to me the task of giving a course of clinical and meteorological lectures in it, which I cheerfully undertake."

